# Micro Germline-Restricted Chromosome in Blue Tits: Evidence for Meiotic Functions

**DOI:** 10.1093/molbev/msad096

**Published:** 2023-04-28

**Authors:** Jakob C Mueller, Stephen A Schlebusch, Yifan Pei, Manon Poignet, Niki Vontzou, Francisco J Ruiz-Ruano, Tomáš Albrecht, Radka Reifová, Wolfgang Forstmeier, Alexander Suh, Bart Kempenaers

**Affiliations:** Department of Behavioural Ecology and Evolutionary Genetics, Max Planck Institute for Biological Intelligence, Seewiesen, Germany; Department of Zoology, Charles University, Prague, Czech Republic; Department of Behavioural Ecology and Evolutionary Genetics, Max Planck Institute for Biological Intelligence, Seewiesen, Germany; Department of Zoology, Charles University, Prague, Czech Republic; School of Biological Sciences, University of East Anglia, Norwich Research Park, Norwich, United Kingdom; School of Biological Sciences, University of East Anglia, Norwich Research Park, Norwich, United Kingdom; Department of Organismal Biology, Evolutionary Biology Centre, Science for Life Laboratory, Uppsala University, Uppsala, Sweden; Department of Zoology, Charles University, Prague, Czech Republic; Institute of Vertebrate Biology, Academy of Sciences, Brno, Czech Republic; Department of Zoology, Charles University, Prague, Czech Republic; Department of Behavioural Ecology and Evolutionary Genetics, Max Planck Institute for Biological Intelligence, Seewiesen, Germany; School of Biological Sciences, University of East Anglia, Norwich Research Park, Norwich, United Kingdom; Department of Organismal Biology, Evolutionary Biology Centre, Science for Life Laboratory, Uppsala University, Uppsala, Sweden; Department of Behavioural Ecology and Evolutionary Genetics, Max Planck Institute for Biological Intelligence, Seewiesen, Germany

**Keywords:** germline-restricted chromosome, genomics, birds, B chromosome, inheritance pattern, synaptonemal complex

## Abstract

The germline-restricted chromosome (GRC) is likely present in all songbird species but differs widely in size and gene content. This extra chromosome has been described as either a microchromosome with only limited basic gene content or a macrochromosome with enriched gene functions related to female gonad and embryo development. Here, we assembled, annotated, and characterized the first micro-GRC in the blue tit (*Cyanistes caeruleus*) using high-fidelity long-read sequencing data. Although some genes on the blue tit GRC show signals of pseudogenization, others potentially have important functions, either currently or in the past. We highlight the GRC gene paralog *BMP15*, which is among the highest expressed GRC genes both in blue tits and in zebra finches (*Taeniopygia guttata*) and is known to play a role in oocyte and follicular maturation in other vertebrates. The GRC genes of the blue tit are further enriched for functions related to the synaptonemal complex. We found a similar functional enrichment when analyzing published data on GRC genes from two nightingale species (*Luscinia* spp.). We hypothesize that these genes play a role in maintaining standard maternal inheritance or in recombining maternal and paternal GRCs during potential episodes of biparental inheritance.

## Introduction

Although a germline-restricted chromosome (GRC) was discovered in the zebra finch (*Taeniopygia guttata*) already 25 years ago (Pigozzi and Solari 1998), it has only recently been recognized that such an extra chromosome is likely present in all songbird species (Torgasheva et al. 2019; Kinsella et al. 2019). Despite its conserved presence throughout ∼50 million years of songbird evolution, the chromosome is highly dynamic in size (Torgasheva et al. 2019; Sotelo–Muñoz et al. 2022) and in genetic content (Kinsella et al. 2019; Schlebusch et al. 2022). Thus, in comparison with the standard set of autosomes and sex chromosomes (together referred to as A chromosomes; “A-chrs” hereafter) which are largely syntenic and collinear (Waters et al. 2021), the GRC is by far the most dynamic chromosome in birds (Bravo et al. 2021; Borodin et al. 2022; Schlebusch et al. 2022), similar to the gene-poor and repeat-rich W chromosome (Zhou et al. 2014; Peona et al. 2021). The size of the GRC can range from a few Mb (micro-GRC) to >150 Mb (macro-GRC) (Torgasheva et al. 2019), and dramatic changes in size can occur over short evolutionary periods (Sotelo–Muñoz et al. 2022). Although the GRC appears to be mostly maternally inherited (Pigozzi and Solari 1998, 2005), there is evidence for occasional paternal inheritance (Pei et al. 2022). The GRC is usually present in two copies during female meiosis and in one copy during male meiosis, but again there are exceptions to this rule (Borodin et al. 2022). For example, in pale martins (*Riparia diluta*) and in sand martins (*Riparia riparia*), both females and males can form bivalent and univalent GRCs (Malinovskaya et al. 2020). In estrildid finches of the genus *Lonchura*, variability in GRC copy number as well as size has been observed within a single male individual (Sotelo–Muñoz et al. 2022). Different hypothetical scenarios have been outlined to explain the non-Mendelian meiotic or mitotic behavior of the GRC, including mechanisms such as selective mitotic or meiotic nondisjunction of chromatids or chromosomes (Borodin et al. 2022).

Little is known about the function of the GRC. In the zebra finch, numerous genes have been identified on the GRC as paralogs of A-chr genes, and many of them seem to be expressed (Kinsella et al. 2019). A recent study on GRCs of two nightingale species (*Luscinia* spp.) suggested that many GRC genes are likely truncated pseudogenes (Schlebusch et al. 2022). A few genes seem to be shared across the GRCs of the currently analyzed songbird species (Schlebusch et al. 2022; F.J.R.-R. & A.S., personal communication), but a large fraction of genes seems to be idiosyncratic for single species. Even among closely related sister species, the shared gene content can be as low as 24% (Schlebusch et al. 2022). It has been speculated that the GRC might be involved in female germline development and gametogenesis (Pigozzi and Solari 1998; Itoh et al. 2009). Indeed, the set of GRC–linked genes that are identified with high confidence in the zebra finch are enriched for functions related to female gonad and embryo development (Kinsella et al. 2019). However, additional genes await discovery or assembly even in this species (Kinsella et al. 2019; Asalone et al. 2021).

Here, we assembled and characterized an avian GRC utilizing long-read genomic and full-length expression data. Such sufficiently complete assemblies are important to add to the currently available short-read GRC assemblies, in order to better understand gene content, structure, and expression on the GRC. Our study species—the blue tit (*Cyanistes caeruleus*)—has been widely studied, including its adaptive history using genome scans (Mueller et al. 2016; Perrier et al. 2020; Mueller et al. 2022). We addressed the following questions. 1) How different is the architecture between paralogous GRC–linked and A-chr genes in terms of degeneration and exon–intron structure? For instance, if genes are being pseudogenized, truncated coding regions and fewer and shorter transcribed isoforms would be expected. The results are relevant to understand the recruitment of genes from the A-chrs and the subsequent functional evolution of GRC genes. 2) Are there enriched functions among the identified GRC genes, and how do these compare with those found in other species? 3) What is the function of GRC genes that show increased expression levels in comparison with A-chr paralogs? These genes will likely contrast with the expected degenerated genes with low expression.

## Materials and Methods

### Cytogenetics

We collected two reproductively active male blue tits near Prague, Czech Republic on May 5, 2022. We dissected the testis and prepared meiotic spreads from testis tissue following Poignet et al. (2021). Then, we immunostained the slides according to Moens et al. (1987) using one or more of the following primary antibodies: 1) rabbit polyclonal anti-SYCP3 (recognizing the lateral elements of the synaptonemal complex), 2) human anticentromere serum CREST (binding kinetochores), and 3) rabbit polyclonal anti-H3K9me3 (detecting lysine methylation on the histone H3 and thus labeling the eliminated GRC when it forms a micronucleus; Torgasheva et al. 2021). Finally, we dried all slides and stained them with DAPI in the mounting medium Vectashield (Vector Laboratories). The quality of the staining allowed the GRC to be categorized as a micro-GRC, although a direct measurement of the size was not possible due to a diffusely stained synaptonemal complex.

### DNA Samples and Sequencing

We collected one reproductively active male blue tit on April 16, 2021, and one female blue tit shortly before laying on April 19, 2022 (complete nest with lining), near Seewiesen, Bavaria, Germany, and flash-froze the testis, ovary, and blood. Samples were kept directly (testis and ovary) or in ethylenediaminetetraacetic acid tubes (blood) at −80 °C. High-molecular weight DNA (testis and blood from the male) and total RNA (testis and ovary) were extracted at the Uppsala Genome Center, Uppsala, Sweden, using the Monarch HMW DNA extraction kit (DNA) and the TRIzol reagent and Phasemaker tube extraction system (RNA). At the same center, the samples were sequenced using long-read PacBio HiFi technology (Sequel II) after library preparation with the SMRTbell Express Template Prep Kit (according to the Iso-Seq protocol for the RNA sample). The testis DNA sample was run on three SMRT cells (5,387,360 HiFi reads with mean length of 18,605 bp), the blood DNA sample on one SMRT cell (1,662,014 HiFi reads with mean length of 20,959 bp), and the testis and ovary RNA samples on two SMRT cells each (7,325,044/9,032,538 HiFi reads with mean length of 3,523/2,940 bp, respectively). The Iso-Seq long-read data were processed using the SMRT Link pipeline and used to characterize and map the full-length transcriptome of the adult testis and ovary tissue.

We estimated expression of all the characterized full-length transcripts using the previously published RNA-seq data from Mueller et al. (2016). In short, we extracted and filtered RNA with poly-A tails from the gonads of five male and five female adult blue tits caught in 2012 at the beginning of the breeding season in Westerholz, Bavaria, Germany, and sequenced these samples using Illumina HiSeq paired-end technology.

### GRC and A-chr Assembly

First, we assembled a new somatic reference genome from the long-read data of the male blue tit blood sample using hifiasm (Cheng et al. 2021). The assembly consisted of 537 primary contigs with a total length of 1.2 Gb and an N50 value of 11.7 Mb. This new reference is less fragmented than the previously published somatic assembly cyaCae2, which used short reads from a blood sample (Mueller et al. 2016), and covers 91% of those scaffolds. The new assembly contains 95.9% of the 10,844 BUSCO marker genes (database passeriformes_odb10) as complete and single-copy genes (Simão et al. 2015).

Second, as described below, we extracted and assembled reads with germline-specific alleles from the testis sequencing data to build a GRC of contigs that were sufficiently different from the A-chr contigs. Our method was designed to identify and compare sufficiently different paralogous genes of the GRC and the A-chrs for downstream differential expression analyses. Thus, we mapped reads of the testis (germline) sample and the blood (soma) sample of the same male individual on the new soma reference genome using minimap2 with permissive parameters (allowing up to 10% sequence divergence and secondary mappings) to allow testis reads of potential GRC origin to map to homologous A-chr regions (Li 2018). Mapping rates were close to 100% for both soma and germline reads. We selected all single-nucleotide variants (SNVs) with soma read depth ≥ 15 and germline read depth ≥ 45 (alleles of depth 1 ignored) using BCFtools mpileup (Danecek et al. 2021). These thresholds were chosen as half of the median read depth of soma or germline, respectively, and resulted in a final set of 6.7 million SNVs. We then compiled germline- and soma-specific alleles in the 41,725 windows of 25-kb size containing a minimum of 20 SNVs using BEDTools map (Quinlan and Hall 2010). We used a custom-made parameter (relspec = [sum of germline-specific alleles − sum of soma-specific alleles] / the number of SNPs) to select the top 1% of windows. This approach separates the two peaks of the relspec distribution (supplementary fig. S1*A*, Supplementary Material online) and results in a micro-GRC (see cytogenetic results) in the downstream assembly. The thus selected 417 windows were defined as regions on which potential GRC reads were mapping (supplementary fig. S1*B*, Supplementary Material online). From these regions, we extracted 8,541 testis reads with germline-specific variants using SAMtools (Danecek et al. 2021) and custom-made R scripts (R core team 2020; supplementary material S1, Supplementary Material online). These reads were then assembled with hifiasm into 60 GRC contigs > 50 kb, with a total length of 6.5 Mb and an N50 value of 100.5 kb (supplementary table S1, Supplementary Material online).

To validate the assembled GRC contigs, we mapped soma and germline reads on the combined genome assembly (A-chr plus GRC) using minimap2 in a stringent setting. Median soma read depth in A-chr and GRC windows of 25-kb size was 30× and 0.4×, respectively, while the median germline read depth on the A-chr and GRC was 88× and 24×, respectively. The higher A-chr coverage in the testis compared with the blood (88× vs. 30×) reflects the higher sequencing effort in the testis sample (median ratio of coverage = 2.87). The difference between the GRC and A-chr coverage within the testis sample (24× vs. 88×) is expected, given that the GRC occurs only as a haploid and only in the spermatocytes of the sampled testis cells. The ratio of the GRC to A-chr coverage (0.27) is consistent with past studies sequencing testes from other songbirds (Kinsella et al. 2019; Schlebusch et al. 2022). We further checked if potential highly similar multicopy regions of the GRC (GRC paralogs) were misassembled or collapsed into single regions. We calculated the corrected germline coverage for each 25-kb window of the GRC (corrected germline coverage = raw germline coverage − [soma coverage * 2.87]). Soma coverage refers to the depth of reads from somatic tissue on the GRC assembly (median of 0.4×; see above), and the factor of 2.87 is the median of the ratio between germline to soma coverage in the A-chr. The copy number is then the corrected germline coverage divided by the median of the corrected germline coverage across all GRC windows (14.47). There are only a few potentially misassembled regions with estimated copy numbers higher than one (supplementary fig. S2*A* and *B*, Supplementary Material online). When we consider the copy number of each window (truncated to an integer), the total length of the GRC would sum up to 9.7 Mb. The main high copy-number region with about 64 copies refers to a region rich with tandem repeats (based on tandem repeats finder analysis, not shown).

### Gene Annotation

We mapped the 400,403 and the 508,427 Iso-Seq full-length transcript sequences from the testis and ovary sample, respectively, as well as the 42,986 National Center for Biotechnology Information (NCBI)-annotated transcript model sequences of the previously published blue tit genome on our assemblies using minimap2. We included the NCBI–annotated transcripts based on RNA-seq data from different tissues including the brain, gonads, and a diverse set of organs, to expand the transcriptome beyond the testis and ovary. We mapped the transcriptome separately on the A-chr and GRC assemblies to characterize paralogous genes with high likelihood both on the GRC and on the A-chr. Transcript sequences that redundantly mapped to the same genomic location were collapsed using tama collapse (Kuo et al. 2020), resulting in 53,120 gene models with 483,842 isoforms on the A-chr and 564 gene models with 5,756 isoforms on the GRC (supplementary material 2, Supplementary Material online). To assign gene symbols (abbreviations for the gene names) to the coding gene models, we mapped all isoform model sequences to the human UniProtKB database of 205,788 protein sequences using diamond in the BlastX ultrasensitive mode (Buchfink et al. 2021). Gene models on the GRC and A-chrs that consistently mapped to the same protein (with the same gene symbol) were considered as paralogs.

### Comparison between GRC and A-chr Genes

Functional enrichment of the protein-coding GRC gene list was tested using PANTHER with complete gene ontology (GO) sets and PANTHER Protein Class annotations (Mi et al. 2019). This list contained 103 different gene symbols, and we used all 11,554 gene symbols annotated on the blue tit A-chr or GRC as background. Additionally, we used the gene list of the assembled GRCs of two nightingale species (*Luscinia megarhynchos* and *Luscinia luscinia*) from a previous study (Schlebusch et al. 2022) to evaluate the generality of the findings.

The sequence completeness of protein-coding genes was evaluated by inspecting the output of the BlastX mapping against the UniProtKB database (see above). We compared the 83 gene symbols of the paralogous genes between GRC and A-chrs by subtracting the respective median values across all isoforms and multiple gene copies within the GRC and A-chrs, respectively.

We quantified the expression of all isoform models in the A-chr and GRC (see above) based on the RNA-seq data of the five testes and the five ovary samples (Mueller et al. 2016). To do this, we used Salmon in the direct read mapping-based mode with the whole genome as a decoy (Srivastava et al. 2020). Transcript per million (TPM) values were summed over all isoforms per gene or gene symbol.

## Results

### GRC Genes and Their Architecture

**Fig. 1.**
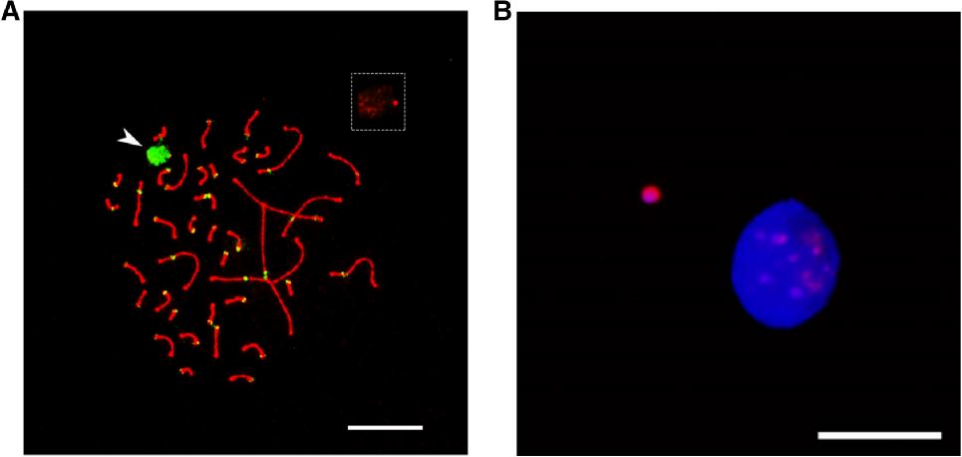
(*A*) Synaptonemal complex spreads of the blue tit immunolabeled with antibodies against SYCP3 (red) and centromere proteins CREST (green). The GRC is indicated with the arrow and also shown in the box at the top right corner without CREST signal. (*B*) Micronucleus containing the eliminated GRC in the blue tit. Micronuclei are immunostained with H3K9me3 antibody (pink), and DNA is counterstained with DAPI (blue). Scale bars represent 10 *µ*m.

**Fig. 2.**
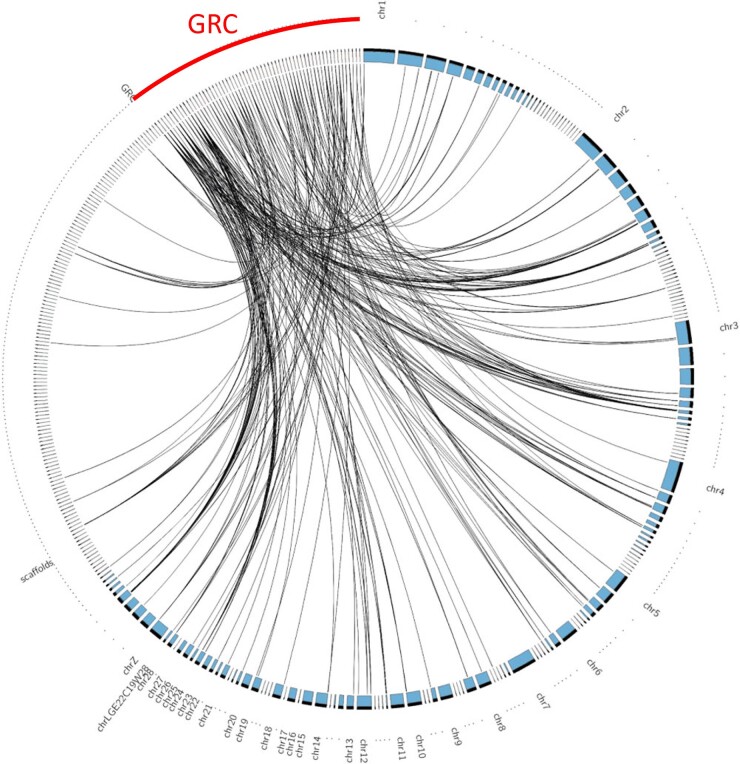
Circos plot of alignment between the 60 GRC contigs and 302 A-chr contigs of cyaCae2 (>50 kb). Alignment blocks of length > 5 kb are linked by a black line. Only the first and largest part of a specific chromosome/scaffold is labeled.

The GRC of the two sampled blue tits has the size of a microchromosome, as shown by cytogenetic techniques (fig. 1). In line with this, we assembled a blue tit GRC of 6.5–9.7 Mb from high-fidelity long reads. The estimated length varies depending on whether copy numbers of assembled regions are included in the estimate. The 60 GRC contigs >50 kb show synteny to many different chromosomes and scaffolds of the previously published soma genome assembly cyaCae2 (Mueller et al. 2016) (fig. 2). As expected, the syntenic GRC regions mostly contain genes (59.3%). Furthermore, specific repeats such as long terminal repeat (LTR) elements and interspersed repeats (as revealed by RepeatMasker; Smit et al. 2015) were more abundant in the GRC than in the A-chr assembly (supplementary table S1, Supplementary Material online).

We characterized 564 genes in the GRC assembly based on full-length transcriptome data, of which 244 showed consistent mapping with one of the UniProtKB proteins and are thus potentially protein coding. The residual set consists of 320 genes that are presumably either noncoding or else unable to match one of the 205,788 known protein sequences, because they are too short or divergent. A gene symbol could be assigned to 164 of the protein-coding genes resulting in 103 different symbols. The percentage of sequence occupied by protein-coding and noncoding genes appears to be higher on our GRC assembly than on the A-chr assembly (supplementary table S1, Supplementary Material online).

**Fig. 3.**
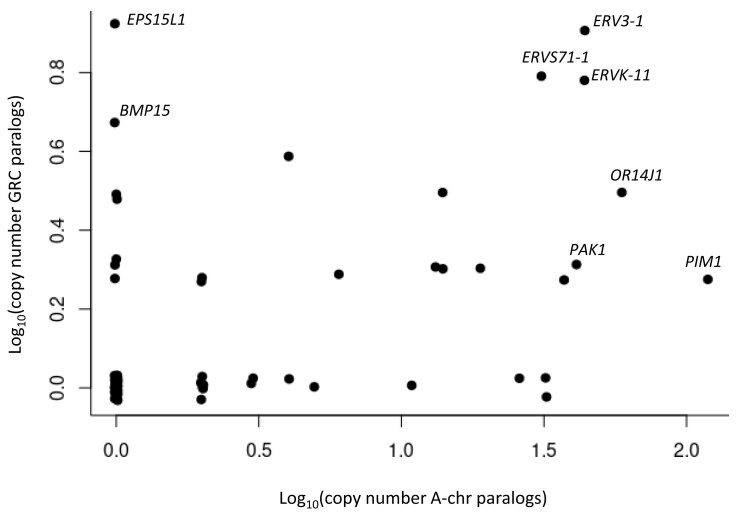
Copy number of GRC genes categorized into the 83 shared gene symbols against copy number of their A-chr paralogs.

We compared pairs of paralogs between GRC and A-chr genes assigned to one of the 83 different gene symbols which occurred in both GRC and A-chr. This set of paralogs consists of 134 annotated GRC and 633 annotated A-chr gene models. The A-chr homologs of GRC genes are distributed across a high number of different contigs or chromosomes, respectively (supplementary table S2, Supplementary Material online). The copy numbers of GRC and A-chr genes of the same symbol are correlated (*r* = 0.35; *n* = 83; *P* = 0.001; fig. 3; supplementary table S2, Supplementary Material online). For instance, genes derived from endogenous retroviruses (*ERVs*) and other known avian multicopy genes (*ORs*, *PAK1*, and *PIM1*) appear in multiple copies both on the GRC and on the A-chrs, whereas other genes like *EPS15L1* and *BMP15* have multiple copies only on the GRC. Most paralogous genes, as represented by GRC gene symbols, have a similar or slightly lower median number of isoforms in the GRC than in the A-chrs with *PIM1*, *PAK1*, and *MROH1* as the strongest exceptions to this rule (Wilcoxon signed rank test: *P* = 0.06; supplementary fig. S3, Supplementary Material online). The median isoform length is often shorter in the GRC than in the A-chrs (Wilcoxon signed rank test: *P* = 0.000001; supplementary fig. S4, Supplementary Material online). In line with this, the percentage of aligned coding sequence of the reference protein is also often smaller in the GRC than that in the A-chrs (Wilcoxon signed rank test: *P* = 0.002; supplementary fig. S5 and table S2, Supplementary Material online). Inspection of the translated sequences of the annotated gene models in the GRC revealed that this was mostly the result of premature stop codons. In addition, comparable exons have similar lengths in GRC and A-chrs, whereas introns are typically shorter in the GRC than in the A-chrs (supplementary fig. S6*A* and *B*, Supplementary Material online).

### Functional Enrichment of GRC Genes

The 103 different gene symbols identified on the GRC are enriched for three hierarchically structured GO cellular component terms related to the synaptonemal complex, in particular to the central element of this complex (table 1). This enrichment is based on the five genes *RNF212B*, *SIX6OS1*, *SYCE2*, *SYCP1*, and *TEX12*. There is one A-chr paralogous gene for each of the genes *RNF212B* and *SIX6OS1* and two paralogous genes for *SYCP1* (supplementary table S2, Supplementary Material online). These four A-chr genes are located on four different contigs and minimal three different chromosomes/scaffolds according to a liftover to the previously published cyaCae2 soma assembly, suggesting that the historical recruitment of the GRC paralogs cannot be ascribed to a single molecular event of duplicating/translocating neighboring A-chr genes. An additional enrichment was found for the protein class “viral or transposable element protein” based on the seven *ERV* genes *ERV3-1*, *ERVK-6*, *ERVK-8*, *ERVK-11*, *ERVK-25*, *ERVPABLB-1*, and *ERVS71-1* (table 1). A linked enrichment of the GO molecular function term “RNA–DNA hybrid ribonuclease activity” was based on the same four *ERVK* genes (table 1).

**Table 1.** Functional Enrichment of the 103 Gene Symbols in the Blue tit GRC.

GO Term/PANTHER Protein Class	FDR	Annotated Genes	Genes in GRC
CC: Central element of synaptonemal complex	0.0035	7	*SIX6OS1*, *SYCE2*, *SYCP1*, *TEX12*
CC: Synaptonemal complex	0.010	29	*RNF212B*, *SIX6OS1*, *SYCE2*, *SYCP1*, *TEX12*
CC: Synaptonemal structure	0.0071	29	*RNF212B*, *SIX6OS1*, *SYCE2*, *SYCP1*, *TEX12*
MF: RNA–DNA hybrid ribonuclease activity	0.045	15	*ERVK-6*, *ERVK-8*, *ERVK-11*, *ERVK-25*
Prot. class: Viral or transposable element protein	0.0000003	18	*ERV3-1*, *ERVK-6*, *ERVK-8*, *ERVK-11*, *ERVK-25*, *ERVPABLB-1*, *ERVS71-1*

Note.—Reference list = all annotated 11,554 gene symbols of combined A-chr + GRC genome. FDR, false discovery rate.

To test whether the detected enrichment of terms on the GRC is a general phenomenon, we additionally analyzed the gene content of the GRC in two nightingale species (Schlebusch et al. 2022). The 84 different GRC gene symbols with >20% of reference coding region found in one of the two nightingale species were functionally enriched against the total of 14,656 annotated gene symbols by only two GO terms, “central element” and “synaptonemal structure” (table 2). The underlying genes *RNF212B*, *SYCE3*, *SYCP1*, and *TEX12* are the same ones as for the blue tit except for *SYCE3* (nightingale only) and *SIX6OS1* (blue tit only).

**Table 2.** Functional Enrichment of the 84 Gene Symbols in the Nightingales' GRC.

GO Term/PANTHER Protein Class	FDR	Annotated genes	Genes in GRC
CC: Central element of synaptonemal complex	0.045	7	*SYCE3*, *SYCP1*, *TEX12*
CC: Synaptonemal structure	0.042	33	*RNF212B*, *SYCE3*, *SYCP1*, *TEX12*

Note.—Reference list = all annotated 14,656 gene symbols of combined A-chr + GRC genome. FDR, false discovery rate.

### Expression of GRC Genes

**Fig. 4.**
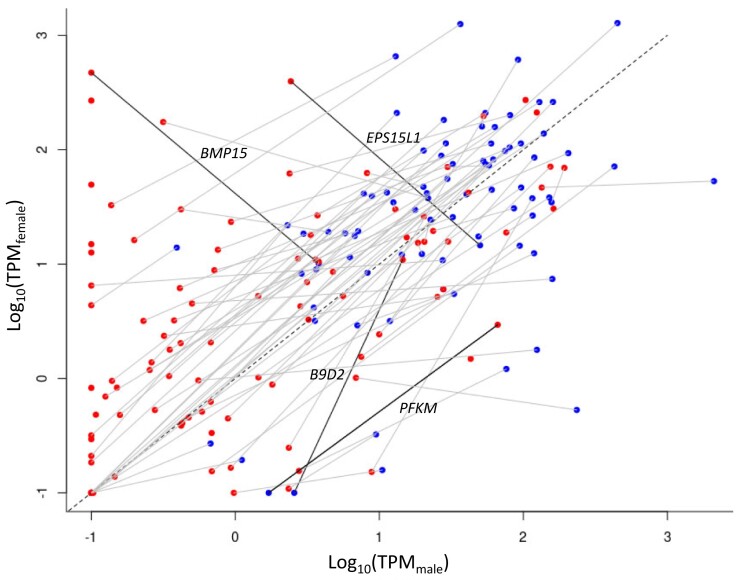
Female against male gonadal expression (log-transformed TPM values) of all A-chr (blue) and GRC genes (red) assigned to one of the 103 GRC gene symbols. Paralogs are linked with a gray line or a black line if TPM is >10-fold in GRC than A-chr homologs in at least one sex. Minimal TPM is set to 0.1.

We found that the protein-coding genes on the GRC in the adult gonadal tissue were mostly expressed at lower levels than the paralogous A-chr genes (fig. 4). This is expected given that oocytes and spermatocytes represent subsets of gonadal cells and that the GRC is haploid in testis. There are only a few exceptions to this pattern (e.g., the genes *BMP15*, *EPS15L1*, *B9D2*, and *PFKM* show >10-fold higher TPM values in GRC than in A-chr paralogs in at least one sex; fig. 4). The majority of GRC genes show a slightly female-biased expression in A-chrs and in the GRC (fig. 4). An extreme example is the gene *BMP15*, which shows a slight female-biased expression on the A-chr but a much stronger female-biased GRC expression. Other genes with strong female-biased expression are *H1-8* and *PIM3* (supplementary table S2, Supplementary Material online). Although the GRC is likely diploid in ovaries, female-biased expression on the GRC is not expected given the lower number of oocytes in the ovary compared with spermatocytes in the testis (supported by preliminary analysis of linked-read DNA sequencing data of ovary and testis samples; J.C.M., personal communication).

## Discussion

To our knowledge, the blue tit GRC assembly described here is the first long-read assembly for any GRC. The underlying HiFi reads should have accuracies >99.5% (Hon et al. 2020). Thus, our study has a similar power to detect germline-specific SNVs as previous linked-read studies but produces a more contiguous assembly due to the substantially longer reads. The length of the assembly is consistent with the GRC being a microchromosome, as revealed by our cytogenetic results, suggesting that we assembled most of the GRC sequence. Our GRC assembly can be considered a core assembly, well suited for direct comparisons between GRC and A-chr genes. However, as our approach to select GRC–specific reads was based on successful albeit permissive mapping of testis reads on a soma assembly, it might miss strongly deviating sequences such as GRC–specific repeats in the GRC. Nevertheless, our approach should include the majority of conserved regions such as the genes. Our method might also miss some regions with very little difference between GRC and A-chr sequences, namely, those that fall below the top 1% threshold of our window selection parameter (see Materials and Methods). Additional alternative sequencing and analysis techniques are essential to reconstruct a complete GRC. A complementary k-mer approach (Rhie et al. 2020) revealed a few additional reads as potentially GRC specific, but these contained mostly tandem repeat sequences (supplementary fig. S7*A* and *B*, Supplementary Material online). Our core assembly, therefore, appeared to be sufficient to reveal major differences in genes located on the GRC and the A-chrs, respectively.

In comparison with their A-chr paralogs, some of the annotated genes on the GRC show signals of pseudogenization in terms of truncated coding sequences, low isoform numbers, short isoforms, and weak expression (Mighell et al. 2000; Cheetham et al. 2020). The expression differences are, however, also expected as a consequence of lower dosage of GRC genes. Testis read coverage on the GRC is about a quarter of that on the A-chr, consistent with the explanation from zebra finch and nightingale studies that only a fraction of cells contains a GRC in gonads and that the GRC is haploid in testis (Kinsella et al. 2019; Schlebusch et al. 2022). Moreover, paralogs of GRC genes are located across most A-chrs, and copy number correlates between GRC and A-chr paralogs, indicating a long history of piecemeal and representative recruitment of GRC genes from all A-chrs (Kinsella et al. 2019; Schlebusch et al. 2022). Despite this tendency for degeneration and representative recruitment, we hypothesize that some gene groups with contrasting patterns might continuously or episodically play an important role in the evolution of the GRC.

The characterized GRC genes are enriched for functions related to the synaptonemal complex. The synaptonemal complex is a multiply interacting protein scaffold that connects homologous chromosomes from end to end during meiotic prophase I and consists of two lateral elements anchored to the chromatin, one central element and transverse filaments spanning the central region and connecting the homologs to each other (Page and Hawley 2004; Cahoon and Hawley 2016; Zwettler et al. 2020). This structure is required for correct pairing of homologous chromosomes, the formation of meiotic crossovers, recombination, meiotic DNA repair, and accurate chromosome segregation. The central and transverse element proteins *SIX6OS1*, *SYCE2*, *SYCP1*, and *TEX12*, which predominantly underlie the detected functional enrichment, should only occur on bivalent chromosome pairs during meiosis I, that is, on the A-chrs during oogenesis and spermatogenesis and on the GRC only during oogenesis under standard GRC inheritance patterns in the germline (Pigozzi and Solari 1998, 2005). Possibly, some basic functions originally coded on the A-chrs could have been transferred to the GRC, in particular for the genes *SYCE2* and *TEX12*, which were exclusively detected on the GRC in our assemblies. Draft assemblies, however, do not allow us to reliably rule out the existence of genes on the A-chrs (as exemplified by the “hidden genes” of GC-rich avian chromosomes; Beauclair et al. 2019; and the recent human telomere-to-telomere assembly; Nurk et al. 2022). Instead of simply leading to a higher gene dosage in the germline through the presence and expression of the GRC, it is more likely that the GRC paralogs of the central element genes confer slightly modified functions specific for the synaptonemal complex of the GRC. This should in particular be true, if the function is “beneficial” for this chromosome. For example, a potential beneficial function could be the stable segregation and inheritance of the bivalent GRC in female meiosis or the facilitation of episodically nonstandard inheritance potentially accompanied with GRC recombination.

Many nonstandard inheritance patterns of the avian GRC have been reported and, given the small sample sizes examined, appear quite frequent among many songbird species (reviewed in Smith et al. 2021; Borodin et al. 2022). If there is a bivalent GRC in the male germline, the central element genes could also play a role here, for example, promoting its segregation or nondisjunction and thus influencing the inheritance of the male GRC. The central element is also essential for recombination between homologous chromosomes (Hamer et al. 2008). Moreover, the other gene detected by the enrichment analysis—*RNF212B*—represents a large-effect locus for recombination rate variation in mammals (Johnston et al. 2020). It is therefore possible that these GRC–specific gene paralogs play a role in promoting or hindering recombination between different GRC types, for example, between maternally and paternally inherited GRCs. Preliminary data from the zebra finch suggest the existence of different, potentially recombining GRC sequences in single populations (Y.P., personal communication). Naturally, novel, coopted, or slightly different functions of GRC–specific genes cannot be excluded and will need further study. SYCP* genes, for example, have been repurposed for kinetochore functions in Kinetoplastida (Tromer et al. 2021). GRC–specific genes potentially have a different functional protein structure than their A-chr paralogs. For example, the three synaptonemal genes with GRC–A-chr paralog pairs differ not only in their untranslated regions (UTRs) but also by 0.4% to 6.1% in their aligned translated amino acid sequence.

Interestingly, the enrichment of synaptonemal complex genes within the GRC is not restricted to the blue tit. Our functional enrichment analysis of the GRC genes identified by Schlebusch et al. (2022) in two nightingale species shows that GRC genes are also significantly enriched for the two GO terms “central element” and “synaptonemal structure.” We therefore hypothesize that regulation in the synaptonemal complex represents a conserved function of the GRC in many songbird species. Independent recruitment in the different species appears to be unlikely, because the A-chr paralogs are located on different contigs/chromosomes suggesting multiple translocation events once in the past. The most recent common ancestor between blue tits and nightingales (and another 3,578 songbird species) presumably lived >27 million years ago (Wong and Rosindell 2022; Oliveros et al. 2019). However, the zebra finch, which is also part of this lineage, potentially lacks these genes in the GRC (Kinsella et al. 2019), suggesting that the synaptonemal function might have been lost in some species later in their evolutionary history. The absence of these genes requires, however, further scrutiny as only a small fraction of the large zebra finch GRC has been assembled (Kinsella et al. 2019; Asalone et al. 2021).

The second enriched group of genes, the family of *ERV*–derived genes, also come in high copy numbers per gene. Again, the nightingales show similarities in this respect (Schlebusch et al. 2022) but not the zebra finch (Kinsella et al. 2019). *ERVs* are common in birds and show a relatively high transcription rate (Bolisetty et al. 2012). Whether these genes are behaving as active transposons/retroviruses or are coopted by the GRC remains to be determined. Some GRCs might simply accumulate ERVs more rapidly than other chromosomes, analogous to the situation on the W chromosome (Peona et al. 2021). In general, a higher density of *ERVs* might have facilitated the transposition or rearrangement of flanking and intervening genomic sequences (Bourque et al. 2018) and therefore might have contributed to the recruitment or excision of sequences during GRC evolution. The LTRs of ERVs contain regulatory elements and also have the ability to modify transcriptional regulation of neighboring genes (Takaya et al. 2021), which might have altered the regulatory network of GRC genes. Interestingly, the enriched *ERV*–derived genes mostly comprise *envelope* genes, which predominantly have been domesticated in the mammalian placenta for novel coopted functions between the fetus and the mother (Haig 2012). Songbird populations appear to vary significantly in the retrovirus lineages transposing among them (Suh et al. 2018; Weissensteiner et al. 2020; Hill et al. 2022), and new *envelope*-containing ERVs are thus continuously accumulating in songbird genomes, with direct or indirect consequences for the GRC.

We annotated many ampliconic gene families on the GRC, some of which also appear as multicopy genes on the A-chr, for example, olfactory receptor genes, *PIM* and *PAK* genes (Kong et al. 2010), and the above-mentioned *ERV* genes. This pattern is not different from randomly recruiting GRC genes from the A-chrs. However, some genes only occur in multiple copies on the GRC. The most prominent ones are the GRC genes *EPS15L1* and *BMP15*. These genes are among those with the strongest overexpression relative to their A-chr paralogs and with the highest female-biased expression among the blue tit GRC genes. Whereas *EPS15L1* appears to be blue tit specific with an unknown function specific for the gonads, *BMP15* was also identified in the zebra finch as the most highly expressed GRC–linked gene (Kinsella et al. 2019). *BMP15* is known to play a role in oocyte maturation and follicular development (Persani et al. 2014). In chicken, *BMP15* is predominantly expressed in the ovary, and its expression is limited to the oocyte (Elis et al. 2007). It has been shown that the recombinant human *BMP15* was able to strongly decrease luteinizing hormone (LH)-induced progesterone production from granulosa cells of large maturing preovulatory follicles (Persani et al. 2014). *BMP15* is also necessary for the maintenance of the female sex in the zebrafish, likely via the regulation of estrogen biosynthesis (Dranow et al. 2016).

In summary, we suggest that the genes shared among the characterized GRCs of songbirds, including *BMP15*, have a high potential to confer basic functions to the GRC. Under predominantly maternal inheritance and female-biased expression of the GRC, these functions would presumably mainly be in the ovaries (adult or embryonic). Such genes might be responsible for the maintenance of the GRC over millions of years. The synaptonemal genes may have been important in shaping the GRC inheritance patterns, either by maintaining the predominant behavior of maternal inheritance or by allowing or facilitating episodes of alternative inheritance patterns.

## Supplementary Material

msad096_Supplementary_Data

## Data Availability

The long-read sequence data (fastq files) and annotated assemblies of the A-chrs and GRC are deposited at the NCBI archive under bioproject number PRJNA925103. Supplementary material 2, Supplementary Material online lists the annotation of all isoform models in bed12 format. The custom-made scripts can be found in supplementary material S1, Supplementary Material online. The previously published blue tit genome browser can be found at http://public-genomes-ngs.molgen.mpg.de.
