# Supplementary material for: Micro Germline-Restricted Chromosome in Blue Tits: Evidence for Meiotic Functions
Source: Mol Biol Evol. 2023 Apr 28;40(5):msad096. doi: 10.1093/molbev/msad096 (PMC10172847; doi:10.1093/molbev/msad096)
Supplement: msad096_Supplementary_Data [file msad096_supplementary_data.zip › JCMuelleretal_GRC_supplfigures1.pptx]

## Slide 1
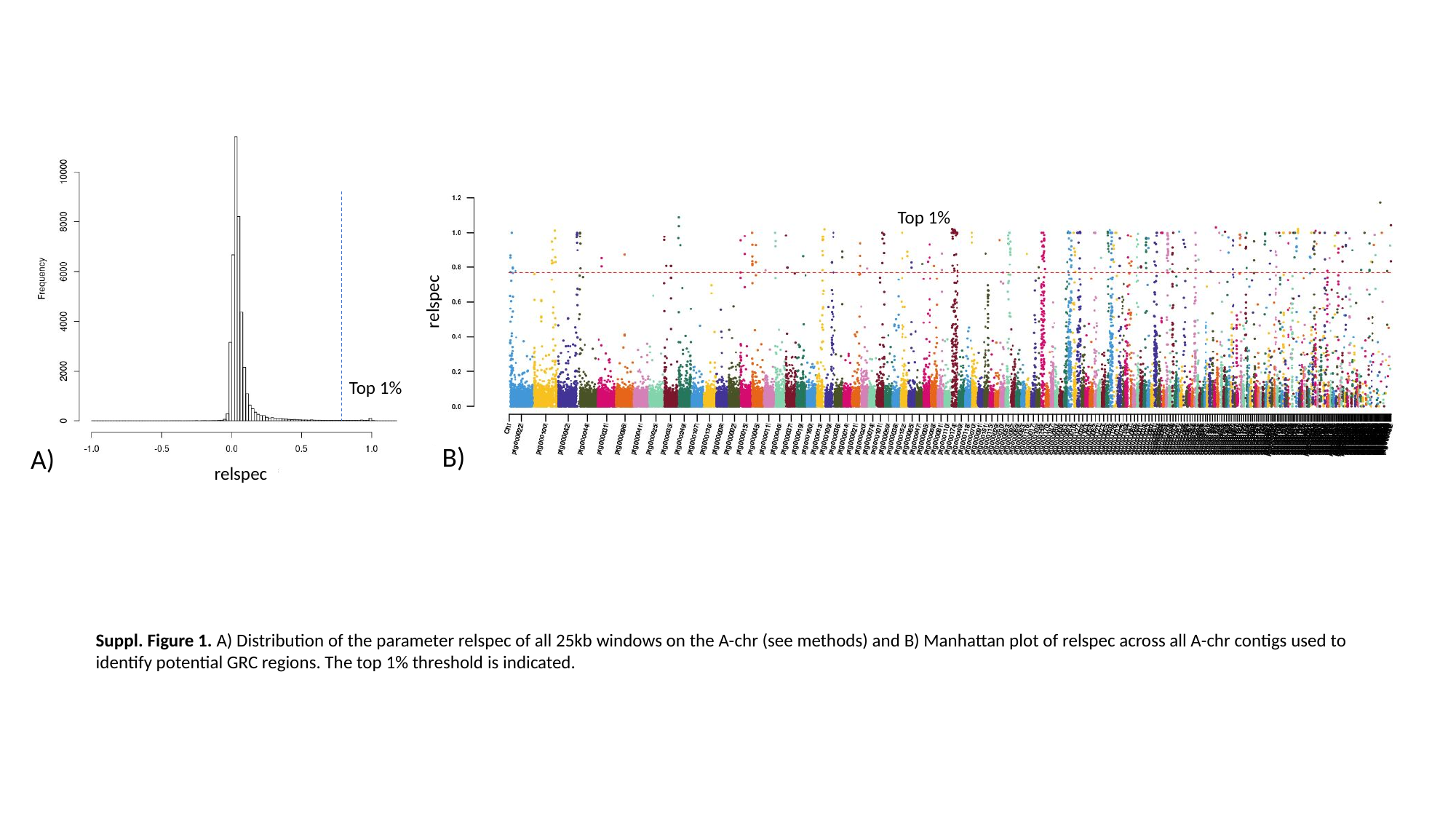

Top 1%
relspec
Top 1%
B)
A)
relspec
Suppl. Figure 1. A) Distribution of the parameter relspec of all 25kb windows on the A-chr (see methods) and B) Manhattan plot of relspec across all A-chr contigs used to identify potential GRC regions. The top 1% threshold is indicated.

## Slide 2
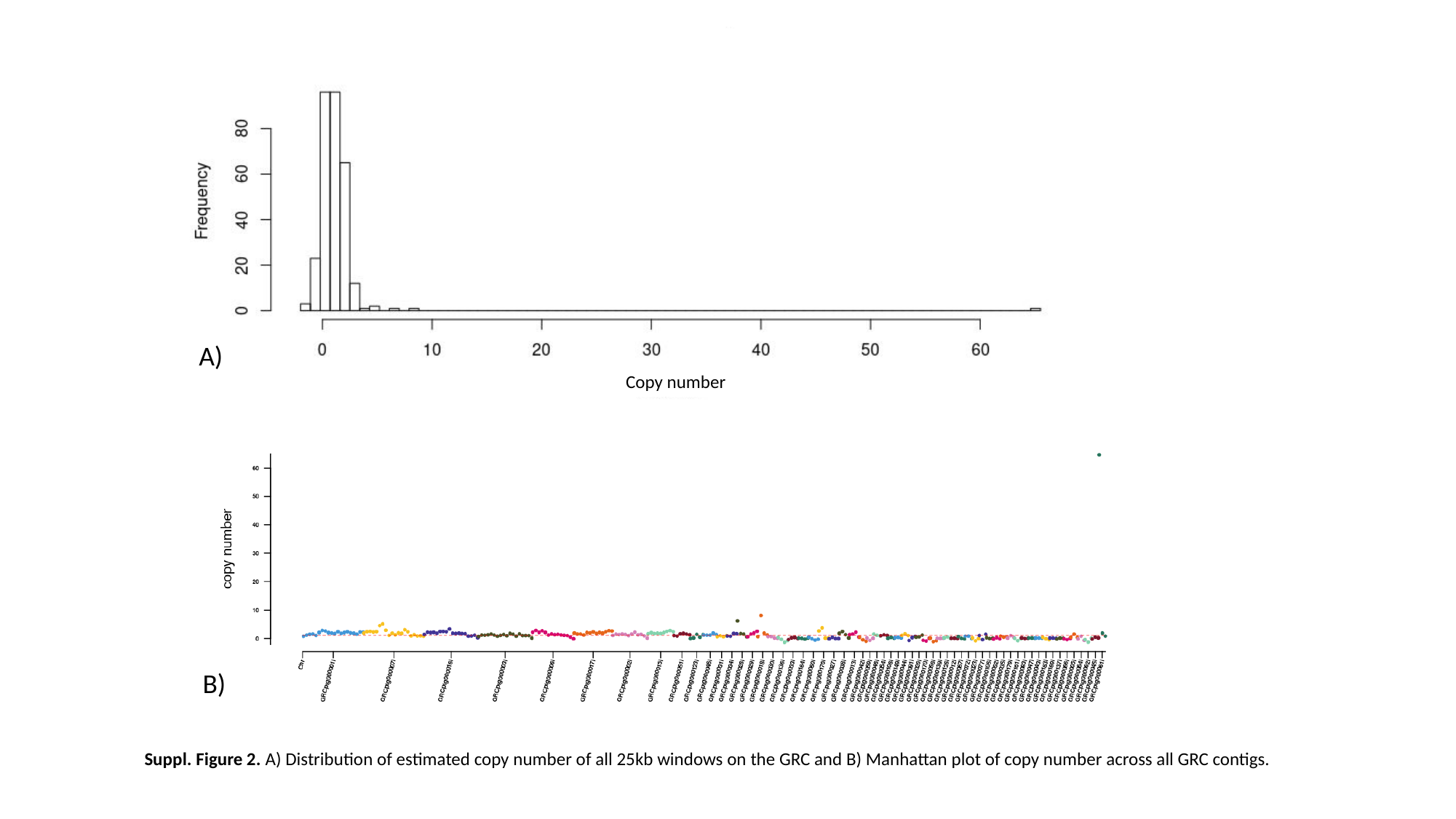

A)
Copy number
B)
Suppl. Figure 2. A) Distribution of estimated copy number of all 25kb windows on the GRC and B) Manhattan plot of copy number across all GRC contigs.

## Slide 3
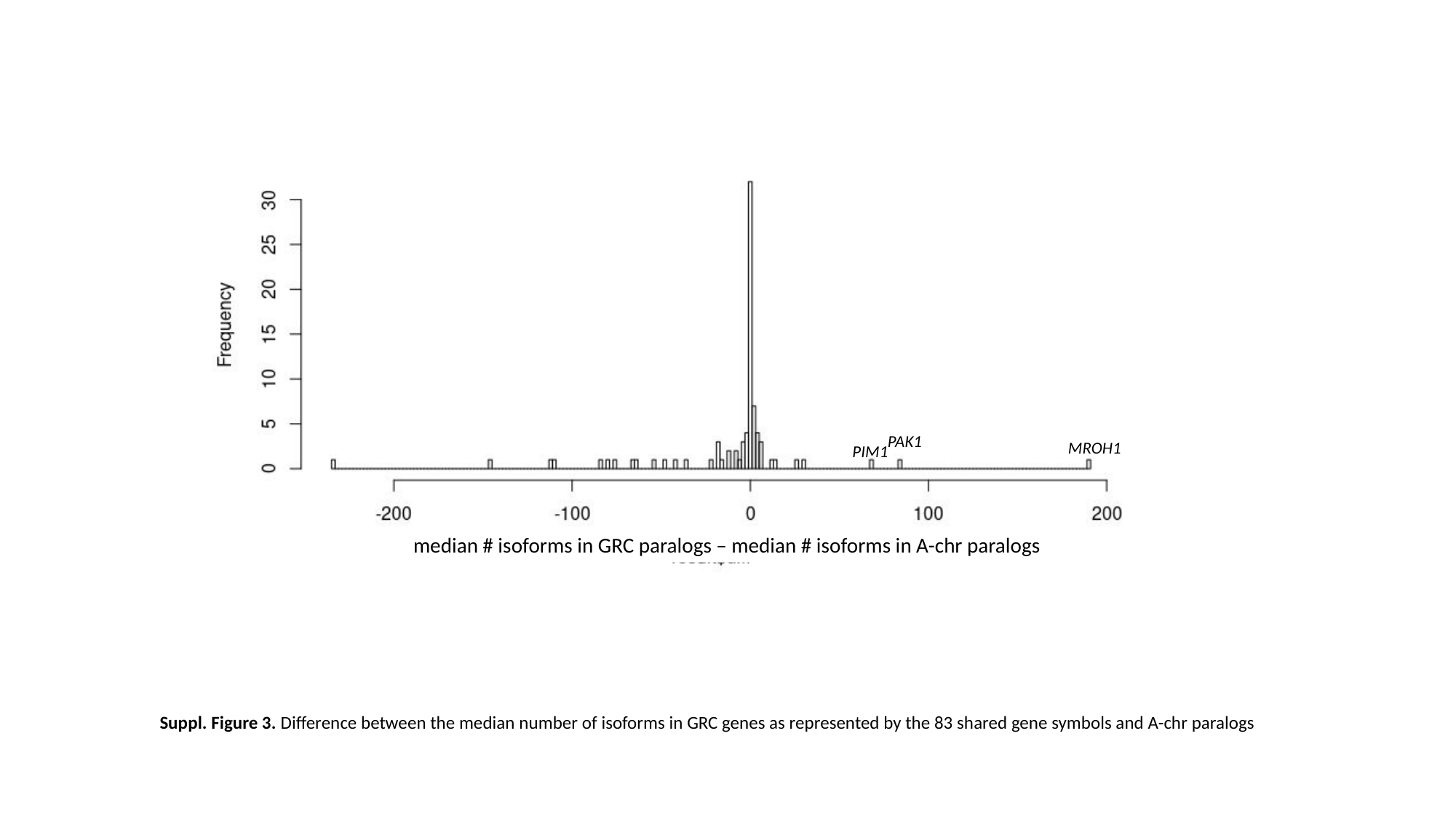

PAK1
MROH1
PIM1
median # isoforms in GRC paralogs – median # isoforms in A-chr paralogs
Suppl. Figure 3. Difference between the median number of isoforms in GRC genes as represented by the 83 shared gene symbols and A-chr paralogs

## Slide 4
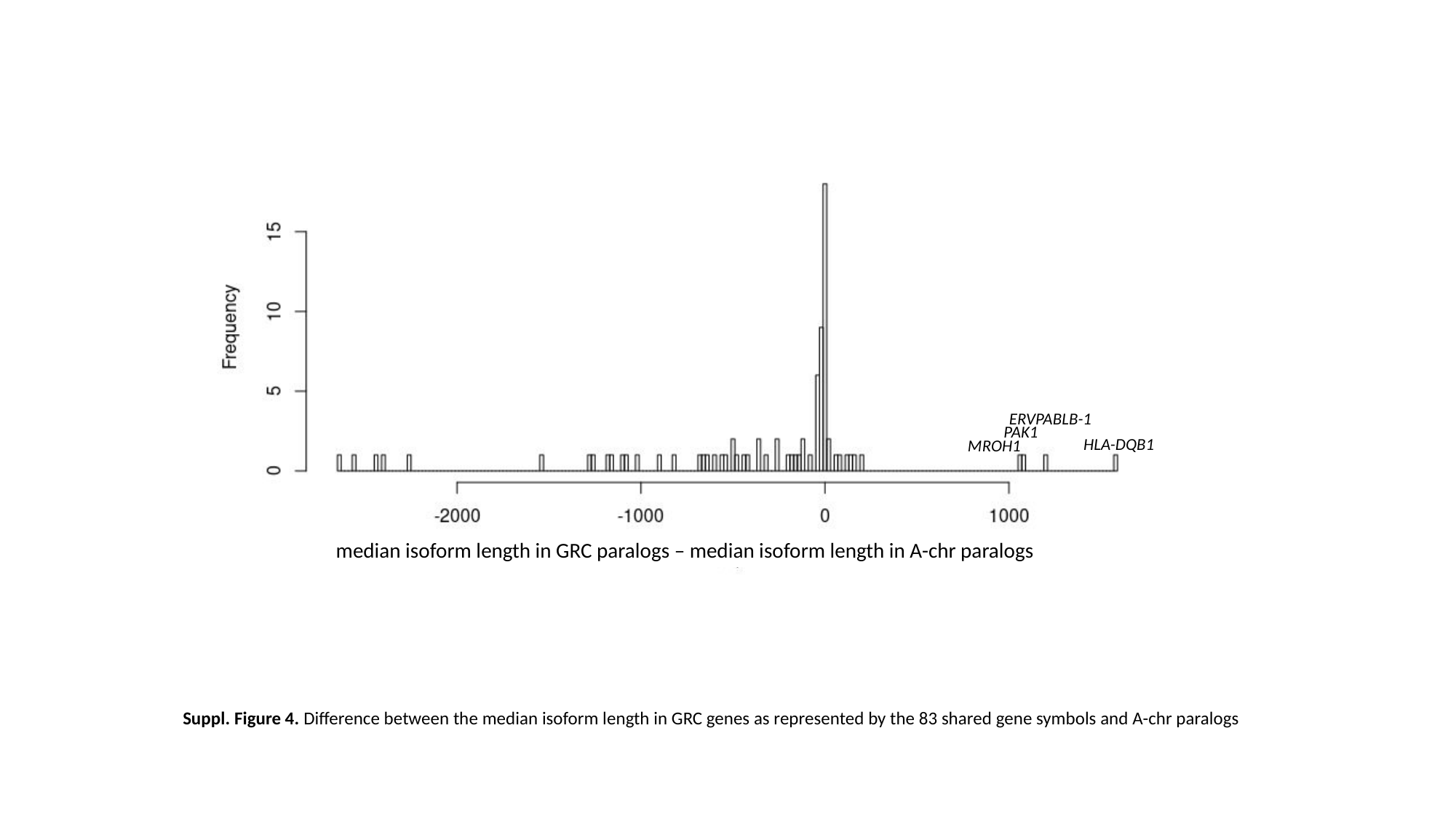

ERVPABLB-1
PAK1
HLA-DQB1
MROH1
median isoform length in GRC paralogs – median isoform length in A-chr paralogs
Suppl. Figure 4. Difference between the median isoform length in GRC genes as represented by the 83 shared gene symbols and A-chr paralogs

## Slide 5
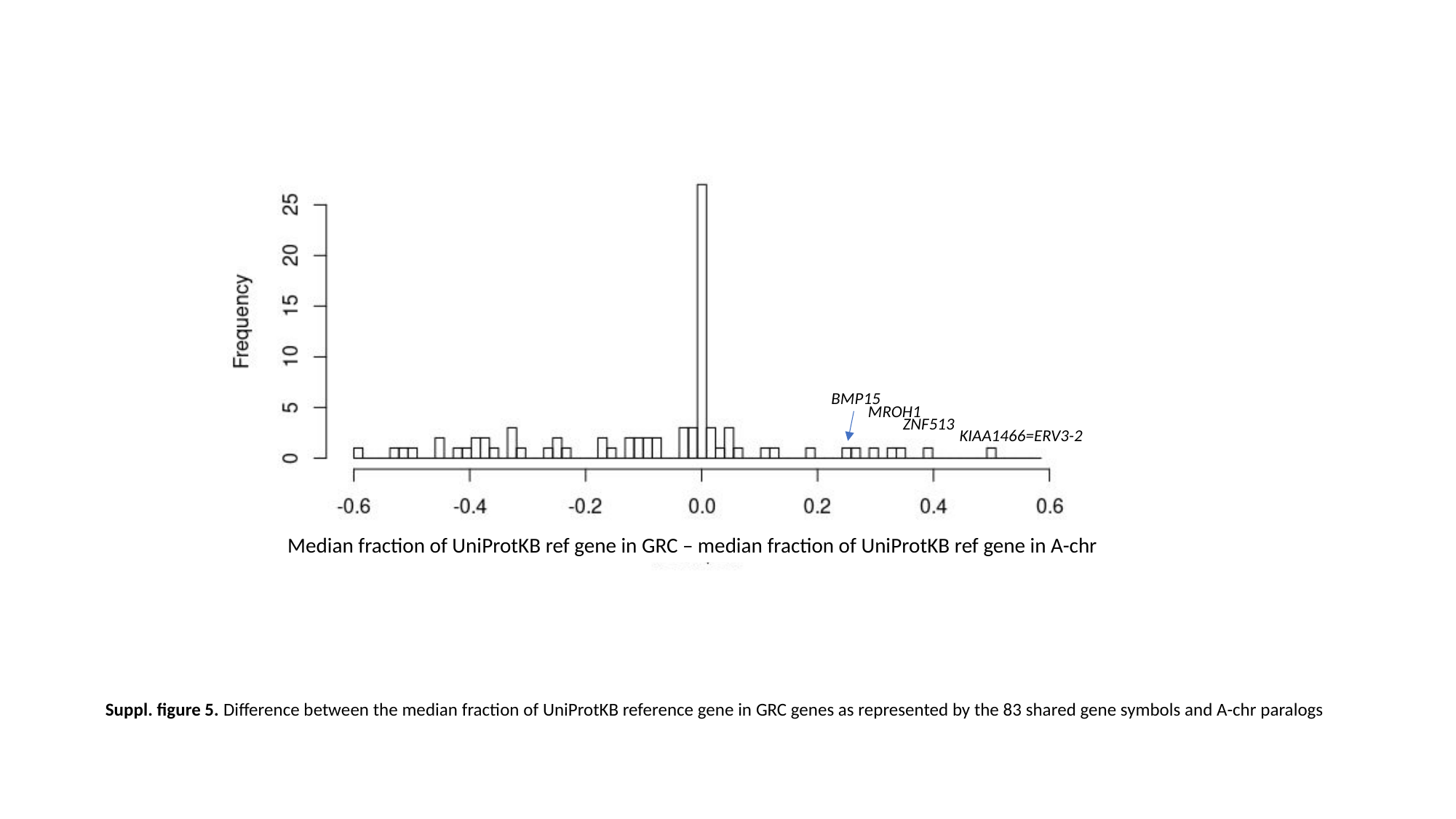

BMP15
MROH1
ZNF513
KIAA1466=ERV3-2
Median fraction of UniProtKB ref gene in GRC – median fraction of UniProtKB ref gene in A-chr
Suppl. figure 5. Difference between the median fraction of UniProtKB reference gene in GRC genes as represented by the 83 shared gene symbols and A-chr paralogs

## Slide 6
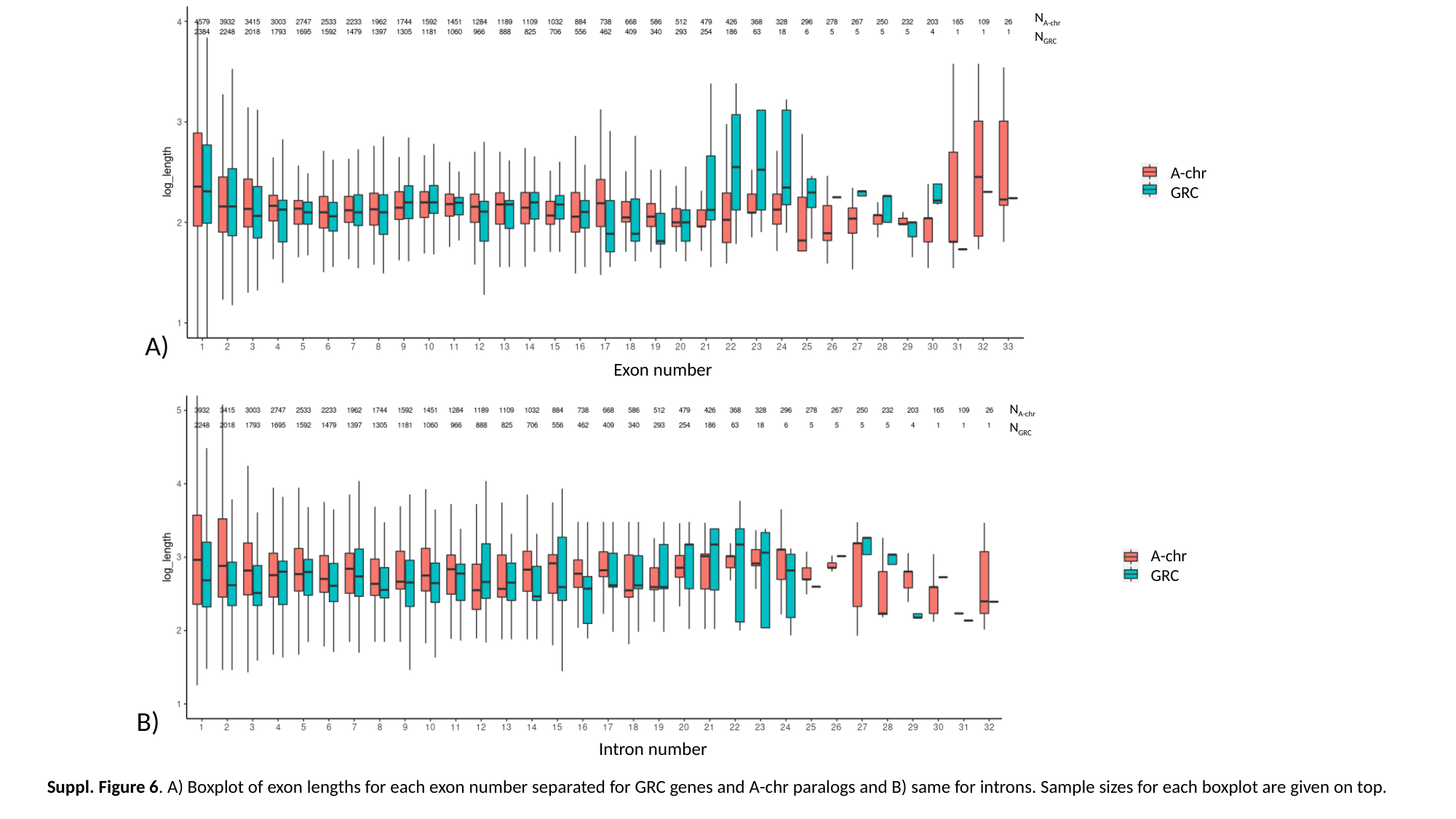

NA-chr
NGRC
A-chr
GRC
A)
Exon number
NA-chr
NGRC
A-chr
GRC
B)
Intron number
Suppl. Figure 6. A) Boxplot of exon lengths for each exon number separated for GRC genes and A-chr paralogs and B) same for introns. Sample sizes for each boxplot are given on top.

## Slide 7
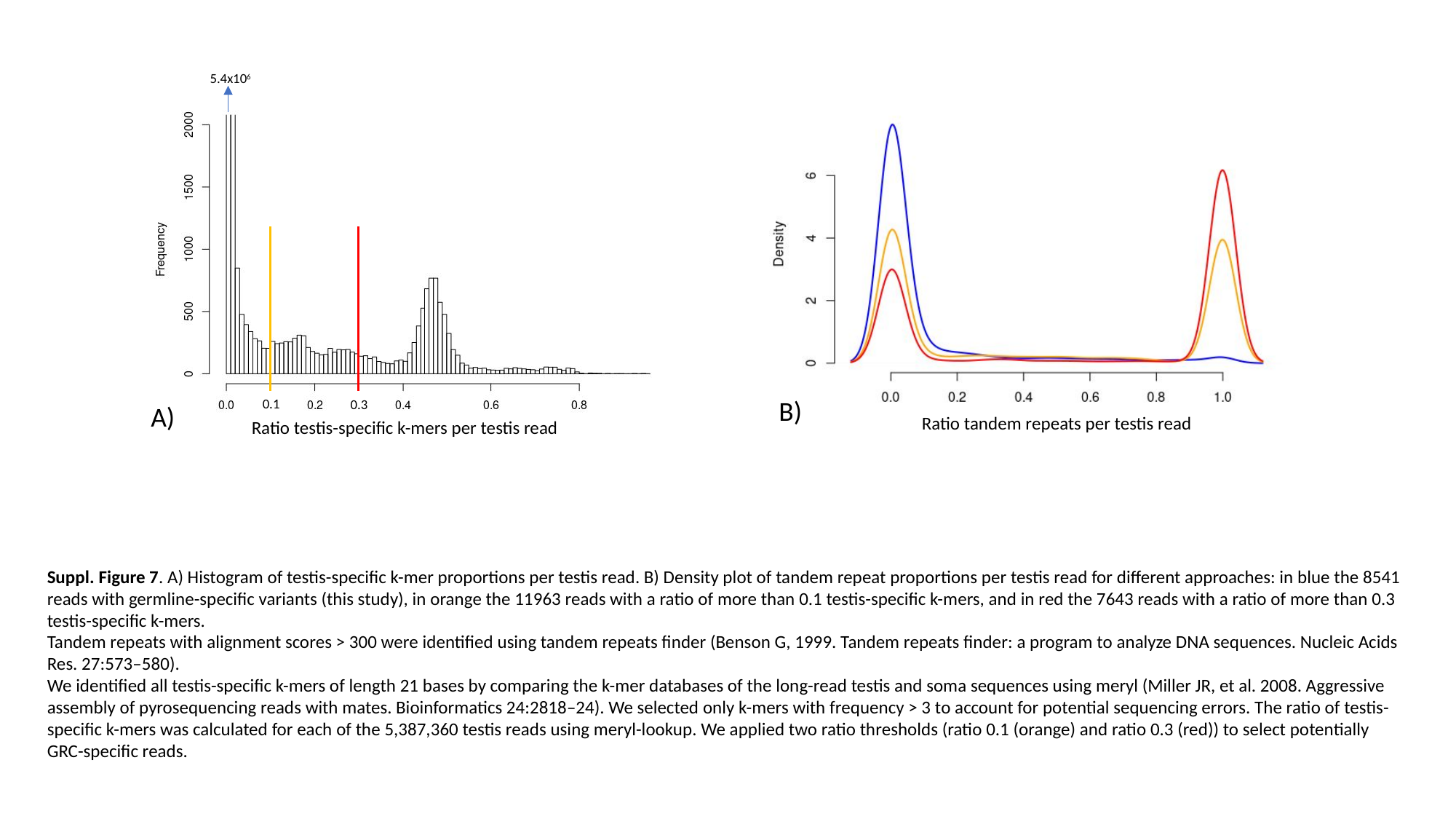

5.4x10^6^
B)
0.1
0.3
A)
Ratio tandem repeats per testis read
Ratio testis-specific k-mers per testis read
Suppl. Figure 7. A) Histogram of testis-specific k-mer proportions per testis read. B) Density plot of tandem repeat proportions per testis read for different approaches: in blue the 8541 reads with germline-specific variants (this study), in orange the 11963 reads with a ratio of more than 0.1 testis-specific k-mers, and in red the 7643 reads with a ratio of more than 0.3 testis-specific k-mers.
Tandem repeats with alignment scores > 300 were identified using tandem repeats finder (Benson G, 1999. Tandem repeats finder: a program to analyze DNA sequences. Nucleic Acids Res. 27:573–580).
We identified all testis-specific k-mers of length 21 bases by comparing the k-mer databases of the long-read testis and soma sequences using meryl (Miller JR, et al. 2008. Aggressive assembly of pyrosequencing reads with mates. Bioinformatics 24:2818–24). We selected only k-mers with frequency > 3 to account for potential sequencing errors. The ratio of testis-specific k-mers was calculated for each of the 5,387,360 testis reads using meryl-lookup. We applied two ratio thresholds (ratio 0.1 (orange) and ratio 0.3 (red)) to select potentially GRC-specific reads.
